# Persistent adrenocortical insufficiency before and after treatment of lymphoma with marked adrenal enlargement — a case series

**DOI:** 10.3389/fmed.2025.1632514

**Published:** 2025-09-03

**Authors:** Yuri Takiyama, Takashi Nanbu, Tasuku Sato, Fumika Maruyama, Yuki Shukuda, Takao Takiyama, Chihiro Sumi, Takeshi Saito, Hiroya Kitsunai, Shuichiro Takahashi, Yumi Takiyama, Hiroshi Nomoto

**Affiliations:** ^1^Division of Endocrinology, Metabolism, and Rheumatology, Department of Internal Medicine, Asahikawa Medical University, Asahikawa, Japan; ^2^Division of Hematology, Department of Internal Medicine, Asahikawa Medical University, Asahikawa, Japan

**Keywords:** adrenal insufficiency, bilateral adrenal tumor, chemotherapy, lymphoma, glucocorticoid replacement therapy

## Abstract

Although lymphoma involving the adrenal gland is uncommon, it is associated with a high incidence of adrenal insufficiency, which may lead to adrenal crisis. The changes in adrenocortical function over the course of lymphoma treatment are not well described. We report three cases with lymphoma with bilateral adrenal enlargement who presented with adrenal insufficiency and had their adrenocortical function monitored during treatment. Case 1 was a 72-year-old man who presented with non-specific symptoms and was diagnosed with lymphoma involving the adrenal glands. Case 2 was a 71-year-old woman who was diagnosed with adrenal lesion of intravascular large B-cell lymphoma. Case 3 was an 84-year-old man diagnosed with primary adrenal lymphoma, presenting rapidly progressing bilateral adrenal tumors. All three were diagnosed with adrenal insufficiency at presentation. Rapid ACTH stimulation test was performed before and after chemotherapy and/or glucocorticoid replacement therapy, and adrenal insufficiency remained in all. Therefore, each required persistent glucocorticoid supplementation despite marked reduction in adrenal lesion. It is important to evaluate adrenocortical function and consider continuing glucocorticoid replacement therapy even after a significant treatment response in adrenal lymphomas.

## 1 Introduction

Approximately half of patients with non-Hodgkin lymphoma (NHL) have extra nodal lesions (1), and tumor involvement of the adrenal glands is observed in approximately 4% of cases on computed tomography (CT) scans (2). NHL is associated with non-specific symptoms such as fatigue, pruritus, weight loss, and fever, and abnormal laboratory tests such as elevated lactate dehydrogenase (LDH) and soluble interleukin-2 receptor (sIL-2R) (3, 4). Approximately 60% of patients with primary adrenal lymphoma present with adrenal insufficiency (5). Clinical signs of adrenal insufficiency include fatigue, abdominal pain, anorexia, hypotension, hyponatremia, and eosinophilia, which appear when 90% of the adrenal cortex has been destroyed (6). Considering that adrenal crisis contributes to increased mortality and symptoms of adrenal insufficiency are generally non-specific, early diagnosis and treatment of adrenal insufficiency is critical. However, the changes in adrenocortical function over the course of the treatment of adrenal lymphoma have not been well described. We present three cases of NHL with adrenal lesions in which we assessed adrenocortical functions before treatment and after significant tumor reduction.

## 2 Case presentations

### 2.1 Diagnosis

#### 2.1.1 Case 1

**Figure 1:**
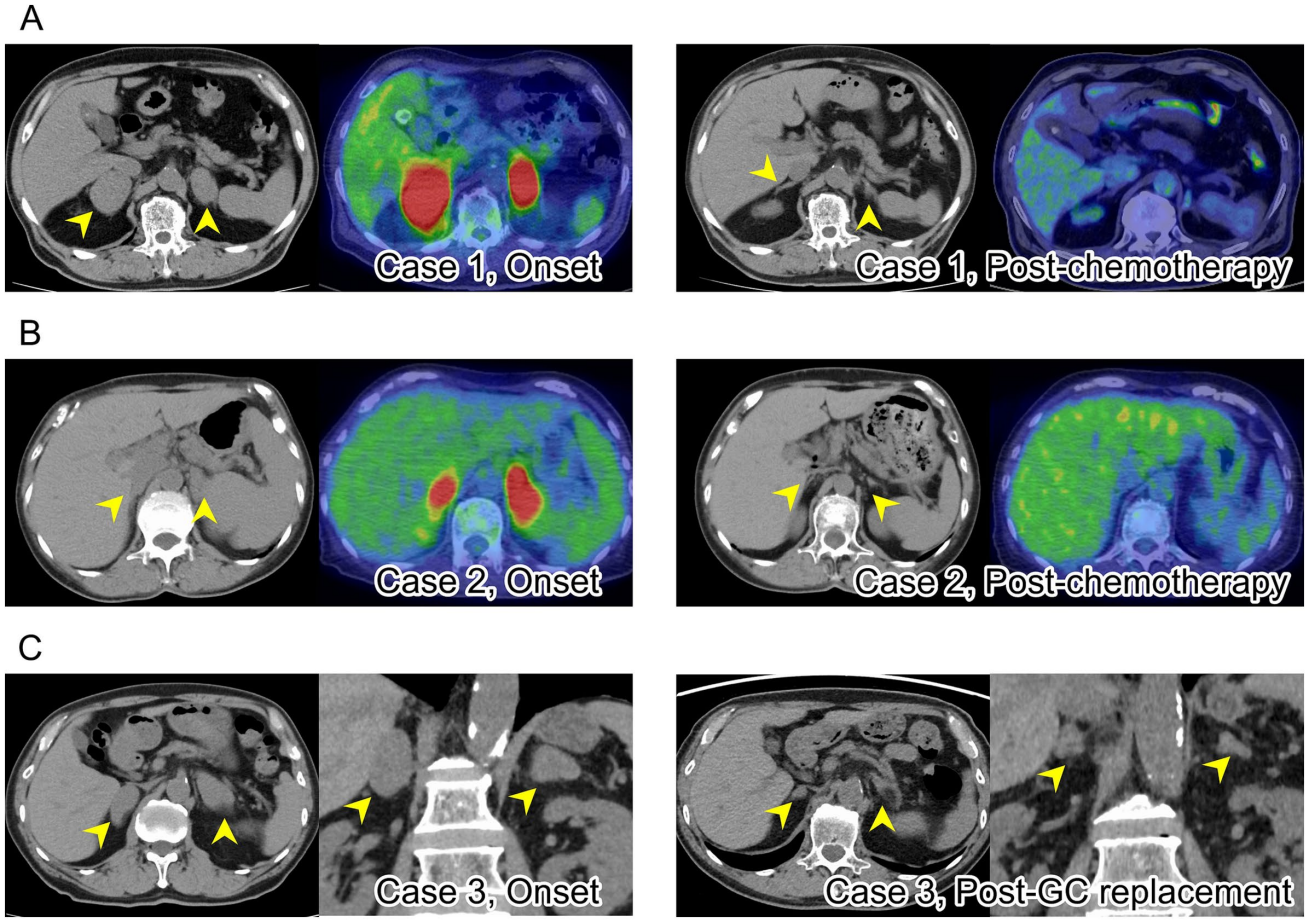
**(A)** Left panel: a plain computed tomography (CT) in case 1 showed bilateral adrenal masses (arrows); ^18^F-fluorodeoxyglucose positron emission tomography (FDG-PET)/CT showed abnormal tracer accumulation in both adrenal glands. Right panel: CT in case 1 after chemotherapy for lymphoma showed shrinkage of the masses (arrows) and decreased FDG accumulation. **(B)** Left panel: CT in case 2 showed bilateral adrenal masses (arrows); FDG accumulation was confirmed in both adrenal glands. Right panel: CT in case 2 after chemotherapy showed shrinkage of the masses (arrows) with normalized FDG accumulation. **(C)** Left panel: CT in case 3 showed bilateral adrenal masses (arrows). Right panel: CT in case 3 after corticosteroid replacement therapy showed shrinkage of the masses (arrows).

A 72-year-old man presented with a 3-month history of fatigue, fever, and weight loss of 5 kg. He was taking mesalazine for his ulcerative colitis. Abdominal CT showed bilateral adrenal tumors (right, 50 × 40 mm; left, 40 × 25 mm; Figure 1A). Superficial lymphadenopathy and signs of Cushing’s disease were not detected on physical examination. Blood test showed anemia and increased concentrations of LDH (258 U/L; reference range 124–222) and sIL-2R (2,342 U/mL; reference range 122–496). His adrenocorticotropic hormone (ACTH) concentration (83.2 pg/mL; reference range 7.2–63.3) was elevated relative to cortisol (10.2 μg/dL; reference range 6.24–18.00), suggesting adrenal insufficiency (Table 1). A rapid ACTH stimulation test (intravenous tetracosactide 0.25 mg) showed no increase in cortisol (Table 2), and replacement therapy with hydrocortisone 10 mg daily was initiated. ^18^F-fluorodeoxyglucose positron emission tomography (FDG-PET) showed increased accumulation of FDG in both adrenal glands (Figure 1A), mediastinal lymph nodes, the L2 vertebrae, and the left ilium. A CT-guided adrenal biopsy showed diffuse large B-cell lymphoma (DLBCL) with expression of CD20, CD10, BCL-6, and MUM-1, but without CD5 expression.

**Table 1:** Endocrine laboratory testing on admission.

Variables	Case 1	Case 2	Case 3	Reference range
Cortisol (μg/dL)	10.8	15.7	6.02	6.24–18.00
ACTH (pg/mL)	83.2	116.0	228.0	7.2–63.3
PRA (ng/mL/h)	0.6	1.5	1.0	0.2–2.3
PAC (pg/mL)	11.1	29.2	9.7	4.0–82.1
DHEA-S (μg/dL)	57	64	27	Male 5–253, Female 7–177
Adrenaline (ng/mL)	≤ 0.01	≤ 0.01	≤ 0.01	≤ 0.17
Noradrenaline (ng/mL)	0.08	0.33	0.23	0.15–0.50
Dopamine (ng/mL)	≤ 0.02	≤ 0.02	≤ 0.02	≤ 0.03
FT3 (pg/mL)	2.57	2.01	2.86	2.30–4.00
FT4 (ng/dL)	1.05	0.99	1.47	0.90–1.70
TSH (μIU/mL)	1.18	3.12	2.31	0.50–5.00
PRL (ng/mL)	4.3	21.9	13.9	Male 4.3–13.7, Female 3.1–15.4
LH (μIU/mL)	7.3	19.7	9.9	Male 2.2–8.4, Female 11.0–50.0
FSH (μIU/mL)	10.1	29.6	11.6	Male 1.8–12.0, Female 26.0–120.0
GH (ng/mL)	0.4	0.8	0.2	≤ 2.47
IGF-1 (SD)	1.3	−0.9	−1.5	−2SD–+2SD

ACTH, adrenocorticotropic hormone; PRA, plasma renin activity; PAC, plasma aldosterone concentration; DHEA-S, dehydroepiandrosterone sulfate; FT3, free triiodothyronine; FT4, free thyroxine; TSH, thyroid-stimulating hormone; PRL, prolactin; LH, luteinizing hormone; FSH, follicle stimulating hormone; GH, growth hormone; IGF-1, insulin-like growth factor-1; SD, standard deviation.

**Table 2:** Serum cortisol values (μg/dL) after the rapid ACTH stimulation test.

Time point	Onset	Post-treatment	Reference range
Case 1			
0 min	10.7	8.5	6.2–18.0
30 min	12.4	9.7	≥ 18.0
60 min	13.0	10.0	≥ 18.0
Case 2			
0 min	9.4	4.9	6.2–18.0
30 min	15.8	7.8	≥ 18.0
60 min	16.6	8.9	≥ 18.0
Case 3			
0 min	6.1	3.2	6.2–18.0
30 min	6.3	3.6	≥ 18.0
60 min	6.7	3.6	≥ 18.0

#### 2.1.2 Case 2

A 71-year-old woman with ulcerative colitis on mesalazine presented for close examination with severe fatigue, fever, and weight loss. Blood test showed anemia, hyponatremia (134 mmol/L), markedly elevated LDH (838 U/L) and sIL-2R (1,871 U/mL), and elevated ACTH (116 pg/mL; Table 2). No physical findings suggested specific endocrine disease and no lymphoadenopathy. Abdominal CT and FDG-PET revealed bilateral adrenal masses (right, 17 × 9 mm; left, 29 × 17 mm) with high-FDG accumulation (Figure 1B). There was no significant increase in serum cortisol in response to ACTH stimulation (Table 2), and hydrocortisone 15 mg daily was initiated for treatment of primary adrenal insufficiency. Random skin biopsy demonstrated the proliferation of large B-cell lymphoma cell in the blood vessels, consistent with intravascular large B-cell lymphoma (IVLBCL). The adrenal lesions were considered to be associated with IVLBCL.

#### 2.1.3 Case 3

An 84-year-old man with autoimmune pancreatitis treated with glucocorticoid therapy 20 years ago and type 2 diabetes presented with rapidly progressing adrenal tumors (right; 45 × 22 mm, left; 45 × 27 mm; Figure 1C). In retrospect, an incidental enlargement of the left adrenal gland (14 × 11 mm) without endocrinological abnormality had been noted the previous year. On admission, his ACTH (228 pg/mL) was elevated relative to cortisol (6.02 μg/dL; Table 1), and a rapid ACTH stimulation test resulted in no cortisol response (Table 2). Adrenal lymphoma was suspected based on his weight loss, elevated LDH (294 U/L) and sIL-2R (3,151 U/mL). Glucocorticoid replacement with hydrocortisone 15 mg daily was initiated to improve his general condition. CT scan showed both adrenal lesions had markedly decreased in size 1 month after starting glucocorticoid replacement (Figure 1C). Histopathology of the biopsy specimen was DLBCL expressing CD20, CD10, BCL-6, BCL-2, and MUM-1. Flow cytometry analysis showed no expression of CD5 on the CD20-positive population. No lymphoma lesions other than those in the adrenal glands were detected; therefore, he was diagnosed with primary adrenal lymphoma.

### 2.2 Treatment and follow-up

Case 1 was treated with six cycles of systemic chemotherapy including polatuzumab vedotin, rituximab, cyclophosphamide, doxorubicin, and prednisolone (Pola-R-CHP) along with intrathecal and high-dose methotrexate to prevent central nerve system relapse. Case 2 was treated with eight cycles of Pola-R-CHP along with intrathecal methotrexate. Six months later, their adrenal glands had decreased in size, and FDG-avid lesion completely disappeared (Figures 1A,B). In case 1, abnormal accumulation in the lymph nodes and bones had also disappeared. Both patients achieved complete remission. Hydrocortisone supplementations were continued after chemotherapy in both cases, and the presence or absence of adrenal insufficiency was confirmed at each outpatient visit using symptoms and blood ACTH and cortisol levels. Twelve months after the end of chemotherapy, adrenal function of case 1 was re-evaluated, and in case 2, her adrenal function was re-evaluated fifteen months after chemotherapy. Cortisol was not elevated after rapid ACTH stimulation test in either patient (Table 2), and glucocorticoid replacement therapy for adrenal insufficiency was continued.

In case 3, the adrenal glands had significantly decreased in size after hydrocortisone supplementation for adrenal insufficiency (Figure 1C). Although systemic chemotherapy was recommended, the patient refused. Therefore, we continued the treatment with low dose corticosteroids. When adrenocortical function was re-evaluated via a rapid ACTH stimulation test about 1 week after the adrenal glands had decreased in size, no increase in cortisol was observed (Table 2), and replacement therapy was continued. Two weeks later, he developed marked hepatosplenomegaly and his general condition deteriorated, which made him ineligible for systemic chemotherapy. He received best supportive care and eventually died of tumor progression.

## 3 Discussion

Among malignant tumors within the adrenal incidentaloma, metastasis from melanoma and carcinomas of the lung, breast, kidney, and colon are most frequent; less than 1% are lymphoma (7, 8). Adrenal insufficiency caused by an adrenal tumor is assumed to result from the destruction or replacement of normal structures by tumor cells (6). In patients with bilateral adrenal tumors, the incidence of adrenal insufficiency is as high as 60% in those with lymphoma, but only 30% in those with metastatic tumors (5, 9). This implies that lymphoma and metastatic tumor infiltrate and destroy the adrenal cells and structures in a different manner. Lymphoma’s effects are presumably driven at least partially via a cytokine-driven paracrine effect within the adrenal microenvironment (5). Considering the high frequency of adrenal insufficiency with adrenal lymphoma and its low median survival of 13 months (10), it is important to evaluate adrenocortical function appropriately in patients with bilateral adrenal tumors.

In our cases, adrenocortical function did not improve despite marked tumor reduction after lymphoma treatment or glucocorticoid replacement therapy. Only a single report has evaluated adrenal function after adrenal lymphoma treatment. As in our case 3, Zaman et al. reported transient regression after short term glucocorticoid administration; although the adrenal enlargement had improved markedly, adrenocortical dysfunction remained and the patient required continued replacement therapy (11). It is presumed that the adrenal cells do not regenerate, and adrenal insufficiency remains even after the lesion shrinks, suggesting that re-evaluation of adrenocortical function and appropriate glucocorticoid supplementation are important even after lymphoma lesion reduction. Additionally, rapid ACTH stimulation test is the most necessary endocrine examination to evaluate adrenal function and can be evaluated with single dose of ACTH. Rapid ACTH stimulation test should always be performed in cases in which adrenal function needs to be evaluated such as these cases.

Interestingly, the patients in cases 1 and 2 had a history of ulcerative colitis. Although immunosuppressive therapy with anti-TNF-*α* and thiopurine in patients with ulcerative colitis has been associated with a two-to-threefold increase in lymphoma incidence, others have reported no such increase (12, 13). Both of our cases with ulcerative colitis had been treated with mesalazine and had no history of immunomodulator use. On the other hand, chronic inflammation can promote the clonal expansion of B and T cells, and autoimmune diseases such as rheumatoid arthritis, systemic lupus erythematosus, and idiopathic thrombocytopenic purpura might be associated with increased risk of lymphoma (14). Therefore, the pathogenesis of ulcerative colitis itself might contribute to lymphoma development.

In case 3, the patient’s general condition worsened rapidly despite a considerable decrease in size in his bilateral adrenal masses after low-dose hydrocortisone administration. Two other similar cases of primary adrenal lymphoma with marked lesion shrinkage after administration of dexamethasone 1 mg daily and hydrocortisone 25 mg daily, respectively, have been reported (11, 15). Glucocorticoids are also used as a lymphoma treatment and Grønning et al. proposed that immature lymphoma cells respond better to glucocorticoids (15). Glucocorticoid replacement therapy is necessary for adrenal insufficiency. However, even small doses may shrink adrenal lesions and make biopsy difficult. Therefore, lesion size should be carefully monitored during replacement therapy and biopsy considered early after its initiation.

## 4 Concluding remarks

Adrenal lymphoma, although rare, can present with adrenal insufficiency, and early diagnosis and intervention are crucial. We experienced three cases of adrenal lymphoma, including lymphoma involvement, IVLBCL, and primary adrenal lymphoma. All three patients exhibited adrenal insufficiency at diagnosis, and their insufficiency persisted even after treatment. Reassessment of adrenal function is necessary after treatment of adrenal lymphoma, even if complete remission is achieved. If functional assessment cannot be performed, glucocorticoid replacement therapy should be continued.

## Data Availability

The original contributions presented in the study are included in the article/supplementary material, further inquiries can be directed to the corresponding author.

## References

[1] Paryani S Hoppe RT Burke JS Sneed P Dawley D Cox RS. Extralymphatic involvement in diffuse non-Hodgkin’s lymphoma. J Clin Oncol. (1983) 1:682–8. doi: 10.1200/JCO.1983.1.11.682, PMID: 6422003

[2] Paling MR Williamson BR. Adrenal involvement in non-Hodgkin lymphoma. Am J Roentgenol. (1983) 141:303–5. doi: 10.2214/ajr.141.2.303, PMID: 6603123

[3] Cheson BD Fisher RI Barrington SF Cavalli F Schwartz LH Zucca E. Recommendations for initial evaluation, staging, and response assessment of Hodgkin and non-Hodgikin lymphoma: the Lugano classification. J Clin Oncol. (2014) 32:3059–67. doi: 10.1200/JCO.2013.54.8800, PMID: 25113753 PMC4979083

[4] Yoshida N Oda M Kuroda Y Katayama Y Okikawa Y Masunari T. Clinical significance of sLI-2R levels in B-cell lymphomas. PLoS One. (2013) 8:e78730. doi: 10.1371/journal.pone.0078730 24236041 PMC3827264

[5] Rashidi A Fisher SI. Primary adrenal lymphoma: a systematic review. Ann Hematol. (2013) 92:1583–93. doi: 10.1007/s00277-013-1812-3, PMID: 23771429

[6] Hintze SC Beuschlein F. Adrenocortical insufficiency after bilateral adrenal hemorrhage due to anticoagulation and chronic immunothrombocytopenia. Endocrinol Diabetes Metab Case Rep. (2024) 2024:24–0034. doi: 10.1530/EDM-24-0034 PMC11623252 39579481

[7] Savoie PH Murez T Rocher L Neuville P Escoffier A Fléchon A. French AFU cancer committee guidelines – update 2024–2026: assessment of an adrenal incidentaloma and oncological management. French J Urology. (2024) 34:102748. doi: 10.1016/j.fjurol.2024.102748, PMID: 39581666

[8] Papageorgiou SG Mavroeidi I Kostakis M Spathis A Leventakou D Kritikou E. Primary adrenal lymphomas with Cushing’s syndrome: two cases with evidence of endogeneous cortisol production by the neoplastic lymphoid cells. J Clin Med. (2023) 12:5032. doi: 10.3390/jcm12155032 37568434 PMC10419581

[9] Redman BG Pazdur R Zingas AP Loredo R. Prospective evaluation of adrenal insufficiency in patients with adrenal metastasis. Cancer. (1987) 60:103–7. doi: 10.1002/1097-0142(19870701)60:1<103::aid-cncr2820600119>3.0.co;2-y, PMID: 3581024

[10] Chen Y Sheng H Hu QF Wang G. Adrenal diffuse large B-cell lymphoma with high PD-L1 expression: two case reports and literature review. J Clin Lab Anal. (2020) 34:e23173. doi: 10.1002/jcla.23173 31903640 PMC7246355

[11] Zaman S Boharoon H Khalid N Marks S Alsafi A Flora R. The vanishing adrenal glands: a transient regression of adrenal lymphoma after a single dose of 1 mg dexamethasone. AACE Clin Case Rep. (2020) 7:109–12. doi: 10.1016/j.aace.2020.11.022 34095465 PMC8053686

[12] Yu J Refsum E Wieszczy P Helsingen LM Perrin V Högdén A. Risk of malignant lymphomas in patients with inflammatory bowel disease: a population-based cohort study. BMJ Open Gastroenterol. (2023) 10:e001037. doi: 10.1136/bmjgast-2022-001037, PMID: 37142293 PMC10163486

[13] Russo MF Diddoro A Iodice A Severi C Gissey LC Casella G. Incidence of lymphomas in inflammatory bowel disease: report of an emblematic case, systematic review, and meta-analysis. Front Med. (2023) 10:1172634. doi: 10.3389/fmed.2023.1172634 PMC10188968 37206474

[14] Zhan L Chen S Liu Y Lu T Yu Z Wang X. Autoimmune disease and risk of lymphoma: analysis from real-world data and Mendelian randomization study. BMC Cancer. (2025) 25:351. doi: 10.1186/s12885-025-13754-4, PMID: 40000981 PMC11863826

[15] Grønning K Sharma A Mastroianni MA Karlsson BD Husebye ES Løvås K. Primary adrenal lymphoma as a cause of adrenal insufficiency, a report of two cases. Endocrinol Diabetes Metab Case Rep. (2020) 2020:19-0131. doi: 10.1530/EDM-19-0131, PMID: 32163909 PMC7077515

